# Induction of oxidative stress biomarkers following whole-body irradiation in mice

**DOI:** 10.1371/journal.pone.0240108

**Published:** 2020-10-01

**Authors:** Tsutomu Shimura, Chinami Nakashiro, Momoka Narao, Akira Ushiyama

**Affiliations:** 1 Department of Environmental Health, National Institute of Public Health, Wako, Japan; 2 Meiji Pharmaceutical University, Kiyose, Japan; Central Research Institute of Electric Power Industry (CRIEPI), JAPAN

## Abstract

Dose assessment is an important issue for radiation emergency medicine to determine appropriate clinical treatment. Hematopoietic tissues are extremely vulnerable to radiation exposure. A decrease in blood cell count following radiation exposure is the first quantitative bio-indicator using hematological techniques. We further examined induction of oxidative stress biomarkers in residual lymphocytes to identify new biomarkers for dosimetry. *In vivo* whole-body radiation to mice exposed to 5 Gy significantly induces DNA double-strand breaks, which were visualized by γ-H2AX in mouse blood cells. Mouse blood smears and peripheral blood mononuclear cells (PBMC) isolated from irradiated mice were used for immunostaining for oxidative biomarkers, parkin or Nrf2. Parkin is the E3 ubiquitin ligase, which is normally localized in the cytoplasm, is relocated to abnormal mitochondria with low membrane potential (ΔΨm), where it promotes clearance via mitophagy. Nrf2 transcription factor controls the major cellular antioxidant responses. Both markers of oxidative stress were more sensitive and persistent over time than nuclear DNA damage. In conclusion, parkin and Nrf2 are potential biomarkers for use in radiation dosimetry. Identification of several biological markers which show different kinetics for radiation response is essential for radiation dosimetry that allows the assessment of radiation injury and efficacy of clinical treatment in emergency radiation incidents. Radiation-induced oxidative damage is useful not only for radiation dose assessment but also for evaluation of radiation risks on humans.

## Introduction

Accidental radiation exposure due to a nuclear accident or terrorism using radioactive materials may cause severe health effects in a large population. Triage of the radiation victims is a major concern in the event of a large-scale radiological emergency. Requirements for preparedness planning for radiation emergencies have been discussed [1]. Dosimetry is an important issue for radiation emergency medicine to provide appropriate strategies for clinical treatment [2]. The cytogenetic assay is the international gold-standard method for biological dosimetry [3]. Biological dosimetry analysis is based on detection of cytogenetic damage in peripheral blood lymphocytes, such as dicentrics, translocations, and micronuclei. The international collaborative network of biodosimetry laboratory services was established around the world for preparedness in case of large-scale radiological incidents. [4, 5]. However, biologically-based dosimeters have some potentially significant limitations. Detection of such damage is time-consuming and not appropriate for rapid assessment. Thus, sample preparation and analysis times must be shortened to meet the needs for emergency assessment. Expertises are required for sample preparation and detecting chromosomal abnormalities. Also the biological responses change in a time-dependent in complex ways and vary among individuals.

Acute radiation syndrome causes vomiting, headache, diarrhea, fever and confusion that appear shortly when whole-body radiation dose is more than one Gy. Radiation-related disease in humans includes bone marrow and gastrointestinal syndromes, cardiovascular disorders and central nervous system syndrome that occur at several weeks after radiation exposure. Implementation of emergency medical treatment for radiation injury can save lives of radiation victims when radiation dose is known [6].

Physical and biological methods are two approaches for radiation dosimetry [2]. Electron paramagnetic resonance (EPR) simply detects radiation-induced radicals in tissue samples such as fingernails, bone, and tooth enamel. This method was used for Japanese atomic-bomb survivors and Chernobyl victims. Further development of individual techniques along with an integrated approach using multiple techniques is required to improve detection limits and improve the time needed for dose assessment in large populations that can be used immediately and retrospectively following external radiation exposure [7, 8].

γ-H2AX a marker of DNA double-strand breaks (DSBs), is a sensitive and powerful radiation biomarker. The γ-H2AX assay useful for clinical evaluation diagnostic computed tomography (CT) scans [9] and pharmacodynamics of anti-cancer drug treatment [10]. Since radiation-induced γ-H2AX foci appear within minutes after irradiation, this assay is useful as a rapid triage tool to prioritize individuals with high levels of DNA damage for follow-up monitoring and treatment [11, 12]. Unfortunately, this marker cannot be used to assess past exposures that occurred a few days prior because γ-H2AX foci rapidly disappear after DNA repair. Other than nuclei, mitochondria are important radiation targets and control redox homeostasis of cells. Biological effects of radiation are primarily caused by reactive free radical-mediated oxidative damage produced by stimulating mitochondrial oxidative phosphorylation (OXPHOS) [13]. Mitochondria release superoxide anions (O_2_^−^), which are subsequently converted to hydrogen peroxide by Mn-superoxide dismutase [14, 15] and further reduced to water by glutathione [16]. However, excess concentrations of reactive oxygen species (ROS) generation leads to oxidative insult to cellular components, including nucleic acids, proteins and lipids. This damage can result in the formation of DNA hydroxylation products, such as 8-hydroxy-2’-deoxyguanosine, and lipid hydroperoxides [16]. Mitochondria are vulnerable to ROS because they are sites of ROS production. Radiation-induced mitochondrial ROS caused oxidative damage in mitochondria in human fibroblasts, resulting in mitochondrial dysfunction [17, 18]. Parkin E3 ubiquitin ligase, is localized in the cytoplasm, is relocated to abnormal mitochondria with low membrane potential (ΔΨm), where it promotes clearance via mitophagy [19, 20]. Nrf2 (NF-E2-related factor 2) transcription factor affects mitochondrial biogenesis through interaction with PGC-1α, a master key transcriptional factor [21]. Nrf2 has cytoprotective roles by controlling the major cellular antioxidant responses [22]. Keap1 facilitates Nrf2 ubiquitination and proteasomal degradation to maintain low levels of Nrf2 under non-stress conditions. Oxidative stress allows ROS to oxidize keap1, which releases Nrf2 that translocates into the nucleus and activates antioxidant response elements (AREs) of several cytoprotective genes such as glutathione transferases (GSTs), γ-glutamylcysteine synthetase (γ-GCS), glutathione peroxidase, heme oxygenase-1 (HO-1), and catalase. Radiation activates this Nrf2-ARE pathway for antioxidant adaptive responses (Fig 5) [23]. Both parkin and Nrf2 have potential as biomarkers for radiation dosimetry.

In the present study, we sought to establish rapid and sensitive methods for radiation dosimetry using animal studies. We found that combining γ-H2AX, parkin, and Nrf2 biomarkers may be useful for extending dose assessment time after emergency radiation incidents.

## Materials and methods

### Irradiation experiments

The mice at six weeks of age were exposed to X-rays generated by a Faxitron CP-160 X-ray apparatus (Faxitron Bioptics LLC, Tucson, AZ, USA) in an acrylic exposure chamber without anesthesia. The 35040 Advanced Therapy Dosimeter (Fluke Biomedical) was used for monitoring radiation dose. X-irradiation was conducted at 160 kV with 0.5-mm Cu and 0.5-mm Al filters. The dose rates were set at 0.6 Gy/min. Biodosimetry data for doses in excess of one Gy are important for triage in emergency radiation accident. Also, biodosimetry data for doses below one Gy is useful to assess the risk of late effects such as cancer and heritable effects. Therefore, we used doses of 0.1, 0.5, 1, 2.5, and 5 Gy for whole body irradiated of mice.

### Animal experiments

All animal experiments were approved by the Animal Ethical Committee of the National Institute of Public Health (NIPH31-008). All experiments were performed in accordance with Japan National Institute of Public Health guidelines and regulations. Male C57BL/6NCrSlc mice at five weeks of age were obtained from Japan SLC (Shizuoka, Japan) and housed under pathogen-free conditions under controlled temperature and humidity, 12h light/12h dark cycle conditions and fed a sterilized pellet diet and water *ad libitum*. Whole body weight of mice was monitored every day except weekend for 7 days after irradiation. The mice which indicate a loss of at least 20% of initial body weight were to be sacrificed and the experiment terminated. Carbon dioxide was used for methods of euthanasia. No mice died prior to application of human endpoints. Mice were anesthetized with an intraperitoneal injection of ketamine (100 mg/kg) and xylazine (10 mg/kg) reconstituted in phosphate-buffered saline (PBS). The cardiac puncture for blood sampling is a terminal procedure. Heparinized peripheral blood samples were collected from hearts in mice which were sacrificed on day 1, 2, and 7. Number of mice in each group was shown in S1 Fig. The number of WBCs was automatically counted with a VetScan HM2 cell counter (Abaxis, Inc., Union City, CA, USA).

### Peripheral Blood Mononuclear Cells (PBMCs) preparation

Heparinized peripheral blood was collected from mouse hearts. PBMCs were isolated using Ficoll‐Paque™ PLUS (Tianjinhaoyang Biological Manufacture Co., Ltd, Tianjin, China) centrifugation. Samples were used for immunostaining or flow cytometry analysis.

### Preparation of blood smears

Blood smears were prepared as described in a previous report [24]. Blood was obtained by nicking a lateral tail vein with a surgical blade. Briefly, a drop of mouse blood (1.5 μL) was mixed with 1.5 μL of phosphate-buffered saline and placed onto dry coverslips. Blood cells were dried for 3 minitues (min) and permeabilized with methanol:acetone (7:3) for six min at −20°C. Cover slips were washed twice with phosphate-buffered saline and samples were fixed with 4% paraformaldehyde (PFA) for 10 min and immunostained with γ-H2AX, parkin and Nrf2 as described below.

### Immunofluorescence

Immunofluorescence staining was performed as described previously [25, 26]. Blood cells were attached onto slide glasses by cytospin centrifugation. Cells were fixed with 4% formaldehyde for 10 min and permeabilized with 0.2% Triton X-100 for five min. Antibodies against γ-H2AX (1:5000 dilution, NB100-384; Novus, Centennial, CO, USA), parkin (1:50 dilution, 14060-1-AP; proteintech, Tokyo, Japan), Nrf2 (1: 100 dilution, ab31163; Abcam, Cambridge, UK) and secondary antibodies conjugated with Alexa Fluor 488 (1:100 dilution, A11034; Molecular Probes, Eugene, OR, USA) were used. Cells were counterstained for DNA with Hoechst 33258 (4 μg/mL in Vectashield mounting medium: Vector Laboratories, Burlingame, CA, USA). Image acquisition and evaluation were conducted with a Keyence BZ-X700 fluorescence microscope (Keyence Corporation, Osaka, Japan) using Hybrid Cell Count software (BZ-II Analyzer; Keyence Corporation). Blood cell number in each sample was indicated in the parentheses in S2 and S4 Figs.

### FACS analysis

PBMCs were stained with parkin antibody in tubes to identify mitochondrial damage and were the transferred into FACS tube. Fluorescence intensity of parkin in PBMCs (total 10,000cells) was quantified using FACScan (Becton Dickinson).

### Mitochondrial membrane potential

PBMCs were stained with JC-1 according to the manufacturer’s instructions (Invitrogen) as we described previously [27]. Cells were incubated in JC-1 for 15min. JC-1 stained cells (total 10,000cells) were quantified using FACScan. The dye can distinguish between mitochondria with low or high membrane potential by formation of JC-1 aggregates (red) and monomers (green), respectively.

### Statistical analysis

Error bars represent the standard deviation. All experiments were repeated at least three times using independent samples. One-way analysis of variance followed by Dunnett’s tests to evaluate significant differences between means of three or more independent groups. Single and double asterisks indicate significant differences between groups of P < 0.05 and P <0.01, respectively.

## Results

### Effects of radiation on hematopoietic tissue

**Fig 1:**
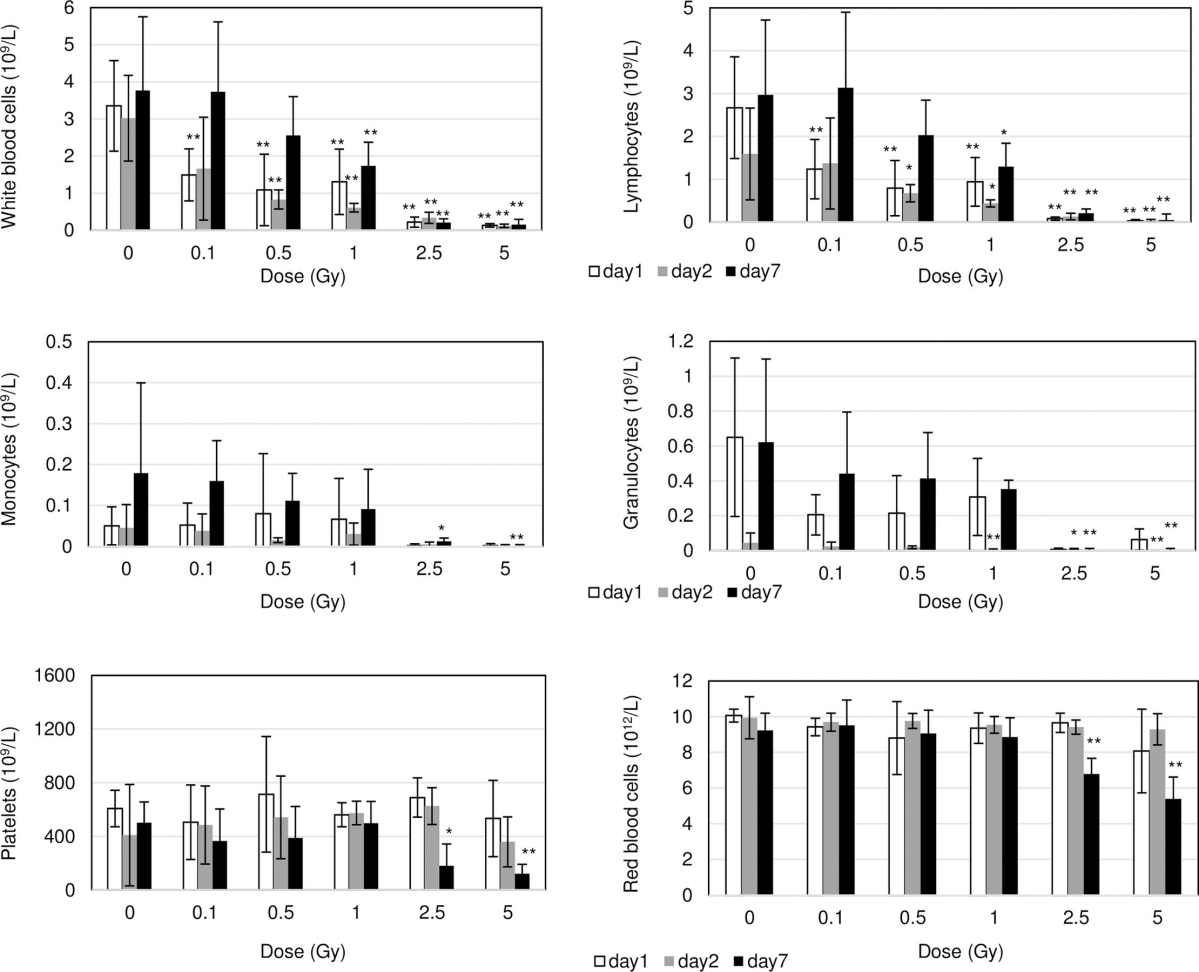
Radiation damage of hematopoietic tissues numbers of cells in WBC, lymphocytes, monocytes, granulocytes, platelets and red blood cells on day1 (open bar) and 7 (closed bar) after irradiation with indicated doses.

To evaluate radiation-induced blood injury, the number of blood cells in irradiated mice was counted at 1, 2 and 7 days after exposure within a dose range from 0.1- to 5 Gy (Fig 1). As compared to non-irradiated control samples, numbers of white blood cells (WBCs), especially lymphocytes, was remarkably decreased on day 1 and day 2 after irradiation. These numbers then recovered to the non-irradiated control levels when radiation dose was below 0.5 Gy. However, low levels did not rebound at doses of >1 Gy by day 7. For monocytes and granulocytes, cell number was significantly decreased on day 2 or day 7 following irradiation compared to non-irradiated control cells. Radiation injury to platelets was noted as a marked decrease in platelets on day 7 after radiation due to relatively long lifetimes of circulating platelets. Reduced red blood cell counts indicated severe damage to bone marrow which led to anemia on day 7 after > 2.5 Gy irradiation.

### Detection of DNA damage, mitochondrial damage and oxidative stress response in irradiated peripheral blood mononuclear cells

**Fig 2:**
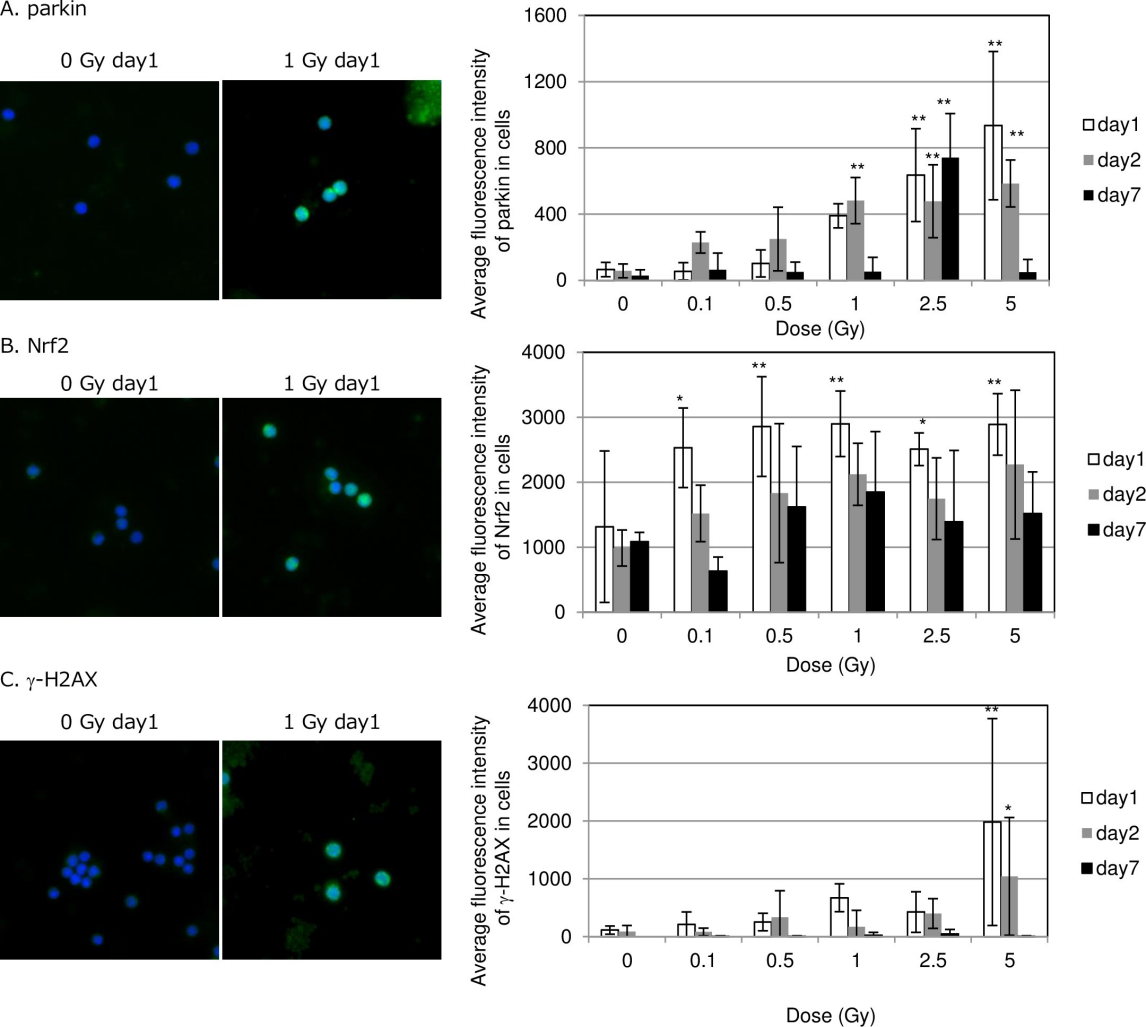
Detection of DNA damage, mitochondrial damage and Nrf2 activation in PBMCs. Image of parkin (A), Nrf2 (B) and γ-H2AX (C) staining in non-irradiated control and irradiated cells at day 1 after irradiation. Magnified image was inserted. Fluorescence intensity of parkin is shown in graph at right.

To develop markers for biodosimetry, mouse peripheral blood mononuclear cells (PBMCs) were isolated from non-irradiated and irradiated mice on days 1, 2 and 7 after irradiation using Ficoll‐Paque™ PLUS (Tianjinhaoyang Biological Manufacture Co., Ltd, Tianjin, China) centrifugation and were attached on slide glasses. We first examined mitochondrial damage in irradiated PMBCs by immunostaining with parkin E3 ubiquitin ligase antibody, which identifies abnormal mitochondria with low ΔΨm (Fig 2A). Parkin staining (green color) was seen in cytoplasm of irradiated PBMCs with >1 Gy irradiation, but not at doses below 0.5 Gy on day 1. Fluorescence intensity of parkin increased in irradiated PMBCs in a dose-dependent manner (Fig 2A left panel). Parkin signals persisted by day 2 and then disappeared by day 7 after exposure to 1 Gy or 5 Gy. Strong Nrf2 staining was evident in nuclei of irradiated PBMCs after exposure to >0.1 Gy on day 1 (Fig 2B). Radiation-induced nuclear Nrf2 staining was not observed on day 2 and day 7 after exposure. We next tested DNA DSBs by immunostaining with γ-H2AX antibody. γ-H2AX foci were induced by exposure to 1 Gy. Increased fluorescence intensity of γ-H2AX staining was observed after exposure to 5 Gy on day 1 and day 2 after irradiation (Fig 2C). Radiation-induced γ-H2AX staining was completely disappeared on day 7 after irradiation.

### FACS analysis for detection of parkin-positive cells

**Fig 3:**
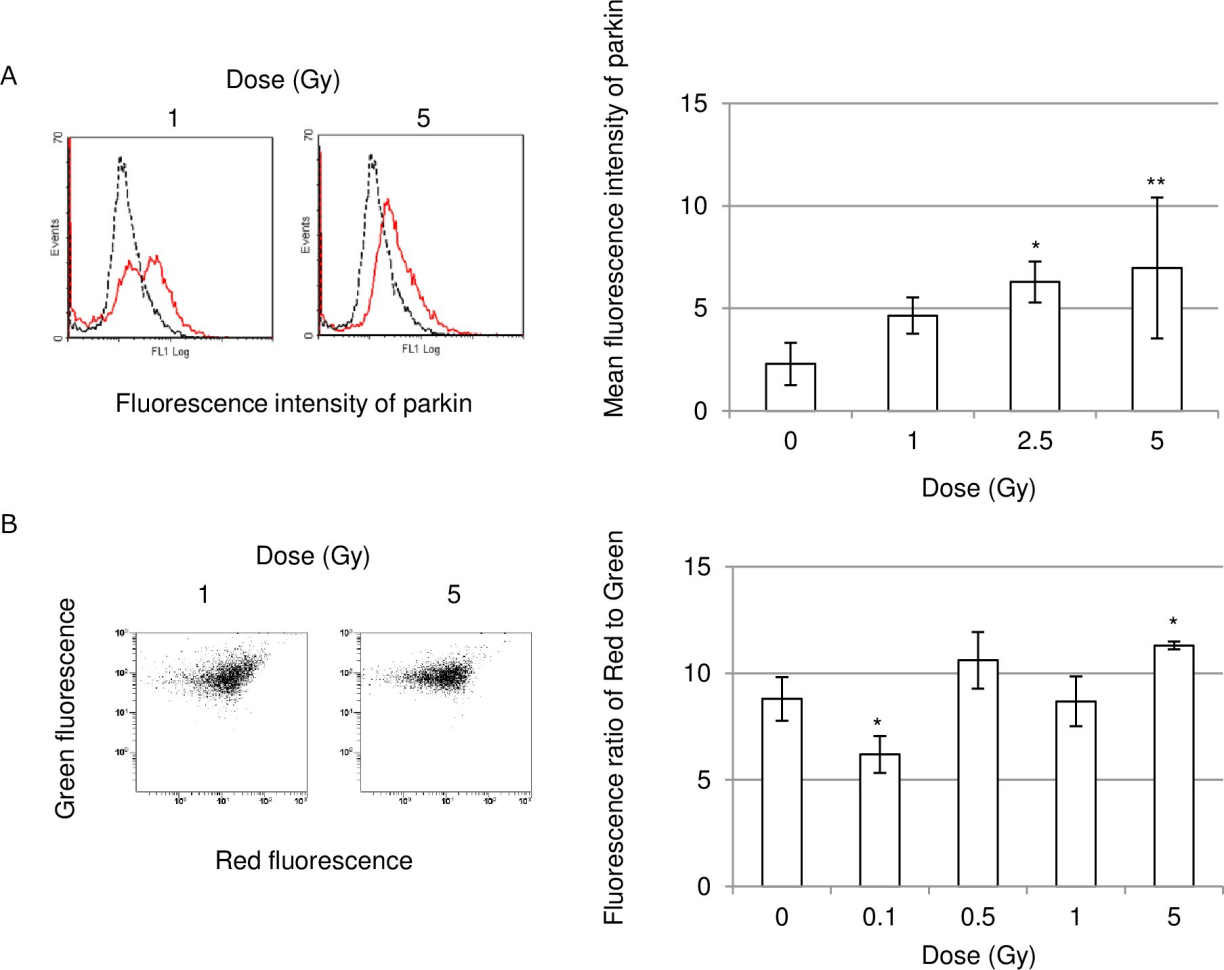
FACS analyses for detecting parkin-staining cells and mitochondrial membrane potential. (A) FACS result for parkin staining on 1 day after irradiation in PBMCs that were either non-irradiated (dotted line) or irradiated (solid line) at the indicated doses. Mean fluorescence intensity of parkin staining is shown in the graph. (B) FACS results for JC-1 staining in non-irradiated cells and 5 Gy-irradiated cells. Blood cells were stained with JC-1 on day 1 after exposure. Asterisks indicate significant differences in FL2/FL1 ratio in irradiated cells as compared with non-irradiated cells.

Fluorescence-activated cell sorter (FACS) analysis can rapidly quantify positive cell populations than image analysis using a fluorescence microscope. We therefore measured the fluorescence intensity of parkin in irradiated PBMCs with FACS. Compared to non-irradiated control groups, increased mean fluorescence intensity values of parkin are detectable after >2.5 Gy exposure of PBMCs (Fig 3A).

The JC-1 probe detects mitochondrial membrane potential (mΔψ) as high and low, as indicated by the formation of JC-1 aggregates and monomers, respectively. The higher value of the JC-1 aggregates/ monomers ratio indicates the higher mΔψ. As well as increased mean fluorescence intensity values of parkin, increased impact on mΔψ was apparent after 5 Gy irradiation in PBMCs (Fig 3A and 3B).

### Detection of parkin, γ-H2AX and Nrf2 in blood smears

**Fig 4:**
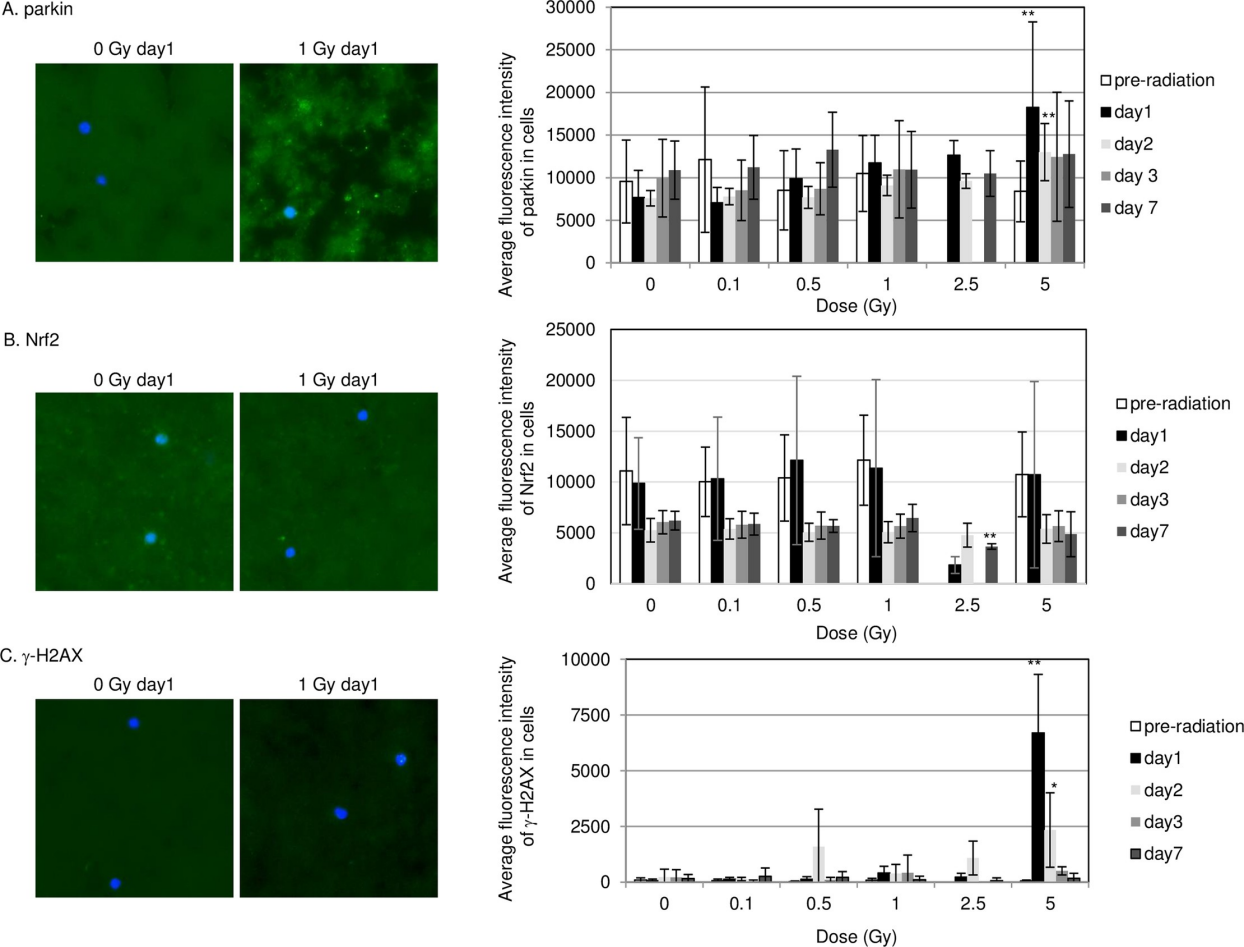
Immunostaining for parkin, Nrf2 and γ-H2AX in blood smear. Image of parkin (A), Nrf2 (B) and γ-H2AX (C) staining in non-irradiated control and irradiated cells at day 1 after irradiation. Magnified image was inserted. Fluorescence intensity of parkin is shown at indicated day in graph at right.

Monitoring radiation responses in mouse blood cells is not possible because of the large amount of blood required for lymphocyte separation with Ficoll density gradient centrifugation. We therefore used a blood drop (less than 3ul) collected from mouse tail at preradiation, and days 1, 2, 3 and 7 following radiation. Blood spread onto glass slides was treated with a stain for parkin, γ-H2AX, and Nrf2. This procedure shortens sample preparing time by omitting the PBMCs separation step. Parkin staining (green color) was evident in cytoplasm of irradiated blood cells (Fig 4A). However, non-specific background signals for parkin were also observed in the background everywhere which do not contain mitochondria. Further, a high background signal was evident for Nrf2 staining in non-irradiated control cells (Fig 4B). Compared to PBMCs isolation methods, background signals in non-irradiated control samples is too high for the detection of radiation-induced signals in blood smear samples. Positive staining for parkin and γ-H2AX staining was only observed by irradiation of white blood cells with 5 Gy (Fig 4A and 4C). Radiation response was not seen in blood smears with Nrf2 staining (Fig 4B).

## Discussion

Dose assessment is critical to identify proper therapeutic strategies and prognoses for radiation victims in a radiation emergency. Radiation biodosimetry techniques using electron spin resonance and biological dosimetry may be useful to establish reliable and sensitive methods for such dose assessment. Vascular endothelial cells and macrophages, as well as hematopoietic stem cells are more sensitive to radiation exposure compared to hematopoietic-supporting stromal cells such as fibroblasts, adipocytes, and endothelial cells [28, 29]. A decrease in blood cell count following radiation exposure is the first quantitative bio-indicator using hematological techniques. Our present data demonstrated that lymphocytes were initially decreased on day 1 after radiation exposure. Irreversibly damaged hematopoietic stem cells no longer generate mature blood cells leading to a reduction in blood cell number on day seven after exposure to >1 Gy. Decrease in platelets and red blood cells were evident on day 7. Several biological markers that show different kinetics for radiation response are needed to extend the measurement period. We used remaining lymphocytes to identify new biomarkers for dosimetry. DNA is the main biological target of radiation and DNA DSBs are visualized by γ-H2AX foci which represent most sensitive endpoint for assessing DSBs. γ-H2AX has potential as a valuable marker for radiation biodosimetry. However, this marker disappears upon repair and is not suitable a few days after radiation exposure. Our data demonstrated that *in vivo* whole-body radiation significantly induced levels of γ-H2AX at 5 Gy dose in mouse blood cells but not at exposures below 1 Gy on day 1 after irradiation.

**Fig 5:**
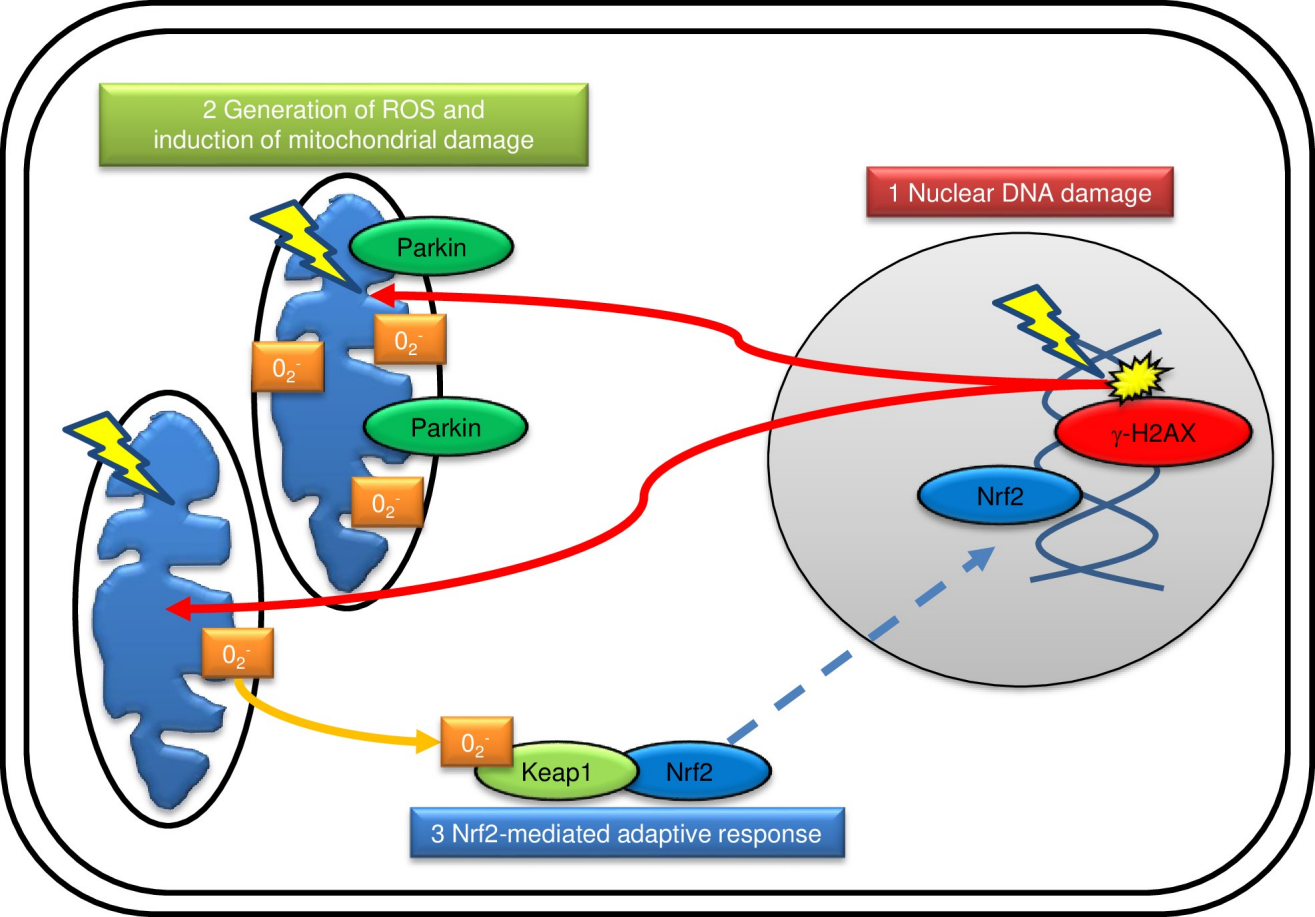
Schematic diagram of sequential induction of γ-H2AX, parkin and Nrf2 following radiation in irradiated mouse blood cells.

Sequential induction of γ-H2AX, parkin and Nrf2 following radiation in mouse blood cells was depicted in diagram (Fig 5). Nuclear DNA damage was immediately induced after irradiation. JC-1 staining revealed that the long-lasting activation of mitochondrial OXPHOS was observed on 1 day in 5 Gy-irradiated PBMCs. The energy supply from mitochondria is required for DNA damage repair that maintains genomic stability of irradiated cells. Stimulating mitochondrial OXPHOS led to ROS generation as a metabolic by-product. Attack of ROS on mitochondria induces oxidative damage as detected by parkin staining and impairs mitochondrial function. This process leads to still more production of ROS [30–32]. Thus, exposure to ionizing radiation leads to oxidizing events that alter *in vivo* redox status. *In vitro* studies using human fibroblasts showed that mitochondrial ROS increases in inverse proportion to the amounts of γ-H2AX foci after irradiation [27]. ROS released from mitochondria appeared 3 hours following irradiation and ROS-mediated mitochondrial damage present at 6 hours and persisted at 24 hours following radiation [27]. ROS also activates Nrf2-mediated adaptive responses. Different kinetics of radiation response were noted among mitochondrial damage, Nrf2-response, and DNA DSBs. *In vivo* radiation oxidative stress was investigated by detecting induction of parkin and Nrf2 in mouse blood cells. Radiation effects were more effectively detected by both oxidative biological markers at lower exposure (1 Gy) than γ-H2AX staining on day 1 after irradiation. Parkin-staining cells were also detected by FACS analysis rapidly to identify radiation victims in a short time compared to image analysis using fluorescence microscopy.

Radiation effects on mouse blood cells can be monitored using a blood drop. This procedure has benefit for preliminary identify radiation victims. However, non-specific signals raise detection limits above limits when using PMBCs. A technique using red blood cell lysis and leukocyte fixation in one step has been shown to be as effective as density gradient centrifugation for preparation of PMBCs for subsequent lymphocyte analysis [33, 34]. Radiation sensitivity may affect various factors such as sex and age at exposure. However, this study only used male mice at six weeks of age. The present manuscript reports the preliminary findings, and contains limitations. Further study is essential to improve biodosimetry techniques to establish simple and rapid procedures for dose assessment.

Since individual radiation sensitivity may influence dose assessment, biodosimetry may be able to screen a highly radiation sensitive population in large nuclear incidents. We previously reported that excessive mitochondrial ROS activates transforming growth factor-beta (TGF-β) signaling, thereby inducing myofibroblast differentiation and facilitating tumor growth through tumor microenvironment formation (TME) formation [35]. ROS-mediated mitochondrial damage is associated with TME *via* fibroblast activation [35]. Consequently, use of oxidative biological markers serves both radiation dose assessment and evaluation of radiation risks for humans. Preliminary dose estimation may help identify radiation victims for early triage. Use of combinations of biological markers that respond with different kinetics to radiation exposure is important for assessment of radiation injury of victims of emergency radiation incidents.

## Conclusion

In conclusion, parkin and Nrf2 are potential biomarkers for use in radiation dosimetry. Identification of several biological markers which show different kinetics for radiation response is essential for radiation dosimetry that allows the assessment of radiation injury and efficacy of clinical treatment after in emergency radiation incidents.

## Supporting information

S1 Fig (XLSX)

S2 Fig (XLSX)

S3 Fig (XLSX)

S4 Fig (XLSX)
